# Fecal glucocorticoid metabolites reflect hypothalamic–pituitary–adrenal axis activity in muskoxen (*Ovibos moschatus*)

**DOI:** 10.1371/journal.pone.0249281

**Published:** 2021-04-14

**Authors:** Juliette Di Francesco, Gabriela F. Mastromonaco, Janice E. Rowell, John Blake, Sylvia L. Checkley, Susan Kutz

**Affiliations:** 1 Department of Ecosystem and Public Health, Faculty of Veterinary Medicine, University of Calgary, Calgary, Alberta, Canada; 2 French Armed Forces Center for Epidemiology and Public Health (CESPA), Marseille, France; 3 Reproductive Sciences Unit, Toronto Zoo, Scarborough, Ontario, Canada; 4 Agricultural and Forestry Experiment Station, University of Alaska Fairbanks, Fairbanks, Alaska, United States of America; 5 Animal Resources Center, University of Alaska Fairbanks, Fairbanks, Alaska, United States of America; Universidade Federal do Parana, BRAZIL

## Abstract

Muskoxen (*Ovibos moschatus)*, a taxonomically unique Arctic species, are increasingly exposed to climate and other anthropogenic changes. It is critical to develop and validate reliable tools to monitor their physiological stress response in order to assess the impacts of these changes. Here, we measured fecal glucocorticoid metabolite (FGM) levels in response to the administration of adrenocorticotropic hormone (ACTH) in the winter (1 IU/kg) and summer (2 IU/kg) using two enzyme immunoassays, one targeting primarily cortisol and the other targeting primarily corticosterone. Fecal cortisol levels varied substantially within and among individuals, and none of the animals in either challenge showed an increase in fecal cortisol following the injection of ACTH. By contrast, two of six (winter) and two of five (summer) muskoxen showed a clear response in fecal corticosterone levels (i.e., maximal percentage increase as compared to time 0 levels > 100%). Increases in fecal corticosterone post-ACTH injection occurred earlier and were of shorter duration in the summer than in the winter and fecal corticosterone levels were, in general, lower during the summer. These seasonal differences in FGM responses may be related to the use of different individuals (i.e., influence of sex, age, social status, etc.) and to seasonal variations in the metabolism and excretion of glucocorticoids, intestinal transit time, voluntary food intake, and fecal output and moisture content. Results from this study support using FGMs as a biomarker of hypothalamic–pituitary–adrenal axis activity in muskoxen, advance our understanding of the physiological adaptations of mammals living in highly seasonal and extreme environments such as the Arctic, and emphasize the importance of considering seasonality in other species when interpreting FGM levels.

## 1. Introduction

Muskoxen (*Ovibos moschatus*) are a taxonomically unique Arctic species and one of the two main ungulate herbivores in the tundra ecosystem [1]. They occupy a wide geographic range throughout the circumpolar Arctic, with endemic populations found in Canada and eastern Greenland, while other populations have been introduced or re-introduced in parts of Quebec, Alaska, Russia, Western Greenland, Norway, and Sweden during the 20^th^ century [2]. Muskoxen are seasonal breeders, with mating occurring in August or September, depending on the latitude, and animals generally reaching sexual maturity at 2 years of age [3]. Climate and other anthropogenic changes are taking place at an unprecedented pace in the Arctic and leading to the occurrence of multiple new stressors, including a higher frequency of extreme weather events, changes in vegetation abundance and diversity, modifications in species distribution and associations, and altered exposure to pathogens [1, 2, 4, 5]. Although muskoxen are well adapted to the Arctic environment, their very low genetic diversity renders them particularly vulnerable to these new and accelerating environmental changes [2, 6]. It is, consequently, becoming crucial to develop and validate reliable tools to monitor their physiological stress response, in order to study the effects that ecological changes are having on individuals and populations, and to identify which factors may be affecting muskoxen the most [2].

The hypothalamic–pituitary–adrenal (HPA) axis is an important mediator of the stress response [7]. A stressor activates the hypothalamus, which then secretes corticotropin-releasing factor and arginine vasopressin to stimulate the anterior pituitary. The pituitary in turn secretes adrenocorticotropic hormone (ACTH) that stimulates the adrenal glands to produce glucocorticoids (GCs), mainly cortisol in muskoxen [8]. Free GCs circulating in the plasma are primarily metabolized by the liver, and the resulting metabolites are excreted via the bile into the intestine, where further metabolism may occur, and some of the metabolites may also be reabsorbed (enterohepatic circulation) [9]. Glucocorticoid metabolites consequently appear in the feces after a species-specific time delay, approximately corresponding to the intestinal transit time, from duodenum to rectum. Fecal GC metabolite (FGM) levels thus are thought to reflect the cumulative secretion and elimination of GCs over several hours to days [10–13].

Fecal GC metabolites have been increasingly and widely used over the past 25 years as biomarkers of the physiological stress response in free-ranging wildlife. Fecal GC metabolite analysis offers the advantage of easy and non-invasive sample collection, absence of capture and handling feedback, and dampening of the pulsatile and diurnal fluctuations in circulating GCs [12, 14, 15].

Glucocorticoids are generally heavily metabolized, which results in multiple metabolites, and usually little to no native hormones, being excreted in the feces [14, 16, 17]. The cortisol and corticosterone enzyme immunoassays (EIAs) commonly used to measure FGMs, therefore, rely mostly on the cross-reactivities of their antibodies to detect this diversity of metabolites [18]. Glucocorticoid metabolism and excretion, the types and proportions of metabolites formed, and consequently, which EIA will be most effective for detecting them, can vary among species (S1 Table) [14]. For example, in Roosevelt elk (*Cervus canadensis roosevelti*), Wasser et al. (2000) demonstrated that the corticosterone antibody used was superior to the cortisol antibody to detect changes in FGMs following a pharmacological challenge [13].

Use of FGMs as a biomarker of HPA axis activity in a novel species, such as muskoxen, requires confirmation that increases in FGM levels reflect changes in adrenal function that can be accurately detected by EIA. Pharmacological challenges involving administration of synthetic ACTH and measurement of the resulting adrenal response have been done across many taxa and are the gold standard for validation [19]. The goal of this study was to validate the use of FGMs as a biomarker of HPA axis activity in muskoxen. More specifically, the objective was to determine whether a single pharmacological stimulation of the adrenal glands (i.e., through the administration of ACTH) was reflected in the FGM levels of muskoxen, measured using two EIAs, one targeting primarily cortisol and the other targeting primarily corticosterone.

## 2. Material and methods

### 2.1 Animals

This study was approved by the Institutional Animal Care and Use Committee, University of Alaska Fairbanks (protocol #1138945), the Veterinary Sciences Animal Care Committee, University of Calgary (protocol #AC16-0259), and the Morris Animal Foundation Animal Welfare Advisory Board. It was done at the Robert G. White Large Animal Research Station at the University of Alaska Fairbanks (USA), where a population of captive muskoxen is maintained for research and teaching purposes.

The muskoxen were housed in groups of two to six individuals based on the established dominance hierarchies and their affinities. They were kept in outdoor pens of mixed pasture dominated by smooth brome grass and boreal forest, varying in size from 0.4 to 11.3 ha. All animals had access to seasonally available forage. They were also provided *ad libitum* fresh grass hay (brome and bluegrass), received a daily pelleted supplement (custom milled by Alaska Pet and Garden, Anchorage, USA), and had access to plain salt blocks. The muskoxen had *ad libitum* access to water as snow in winter and in troughs throughout the rest of the year.

All animals were accustomed to routine movement from their pens to a smaller handling area, which led to a modified bison standing squeeze chute with a load scale for measures of body mass (± 1 kg).

### 2.2 ACTH challenges and fecal sampling

We did two ACTH challenges, one in winter, and the other in summer, 2018. The animals were part of a broader study aiming to validate the use of hair cortisol as a biomarker of HPA axis activity in muskoxen, which involved hair sampling at the time of the injection both in the winter and summer [20]. The muskoxen were randomly allocated to the control (saline) and ACTH groups as part of this broader study and were chosen to be sampled for feces based on the ease and safety of sample collection: six animals were thus sampled in the winter and seven in the summer, all consisting of intact males and non-pregnant females, with some of the muskoxen switching experimental group for the summer challenge or being included in only one of the challenges (Table 1).

**Table 1:** Identification (ID), sex, age, and experimental group of the animals included in the winter and summer ACTH challenges and sampled for feces.

Animal ID	Sex	Winter challenge		Summer challenge	
		Age (years)	Experimental group	Age (years)	Experimental group
MX-738	M	0.75	Not included	1.25	ACTH
MX-740	F	0.75	ACTH	1.25	Control
MX-741	M	0.75	ACTH	1.25	ACTH
MX-620	M	1.75	ACTH	2.25	Not included
MX-621	F	1.75	ACTH	2.25	ACTH
MX-597	F	2.75	Not included	3.25	ACTH
MX-283	F	5.75	ACTH	6.25	Control
MX-1169	F	6.75	ACTH	7.25	ACTH

#### 2.2.1 Winter challenge

On February 5^th^, 2018, six muskoxen (Table 1) received a 1 IU/kg intramuscular (IM) injection of Corticotrophin (Wedgewood Pharmacy, Swedesboro, NJ, USA, a long-term release gel formulation of ACTH; concentration of 80 IU/ml) in the shoulder. This dosage was chosen based on similar studies done in other ungulate species [13, 21–23]. All injections were given between 8:20 and 11:20 AM. Since this challenge served as a pilot study to test the ACTH dose and winter field conditions rendered sampling challenging, no control animals were included.

February is one of the coldest months in Fairbanks with an average low temperature of –25°C, an average high temperature of –12.2°C, 120 h of sunshine, an average precipitation of 11 mm, and an average snowfall of 20.3 cm [24].

Fecal samples were collected from the six animals 0–2.6 (referred to as time 0), 2.7–7.4, 22–24.6, 29.8–33, 45.3–48.2, 52.5–55.3, 68.5–74.3, and 92.5–98.8 h after the ACTH injection. Some of the muskoxen were not sampled at each time-period because of biological challenges (i.e., animals did not defecate during the time-period) and technical constraints (i.e., small number of workers and limited daylight), while others were sampled several times. To collect fecal samples, animals (all well habituated to humans) were observed from a distance until they defecated. They were then approached slowly and the entire fecal pile was collected with gloves from the ground. Feces were immediately placed in a Whirlpack® and then in a cooler with icepacks for a maximum of 4 h before being stored frozen at −20°C. Samples were shipped frozen to the Endocrinology Laboratory of the Toronto Zoo for FGM analysis, no later than 3 months’ post-collection.

#### 2.2.2 Summer challenge

On July 23^rd^, 2018, five muskoxen received a 2 IU/kg IM injection of Corticotrophin in the shoulder. Two control animals were administered an equivalent volume of physiological saline (0.9% of sodium chloride), IM (Table 1). The dose of 2 IU/kg, twice that of the previous challenge, was given because there were some non-responders, based on FGM analyses, during the winter challenge (see Results). All injections were given between 9:00 AM and 12:00 PM.

July is the warmest month in Fairbanks with an average low temperature of 11.3°C, an average high temperature of 22.8°C, 274 h of sunshine, and an average precipitation of 55 mm [24].

Feces, sampled as indicated for the winter challenge, were collected the day before (referred to as time 0), and then 6.7–8.4, 22–24.9, 29–33.2, 45.5–48.5, 53.3–57.4, 68.8–72.3, and 90.8–96 h after the ACTH/saline injection from the five ACTH-injected and two control animals.

### 2.3 Hormone analyses

All hormone analyses were done at the Endocrinology Laboratory of the Toronto Zoo. Both cortisol and corticosterone EIAs were used to quantify FGMs in this study. To validate the EIAs, immunological similarities between the standard and sample hormones were evaluated by assessing parallel displacement between the standard curve and a serial dilution of a pooled muskox fecal extract. Sample dilution was selected based on 50% binding of the pooled sample curve. The recovery of exogenous hormone added to pooled muskox fecal extracts was also tested. The percentage recovery was calculated as (amount observed/amount expected) × 100, with the amount observed corresponding to the value obtained for the spiked sample minus the amount of endogenous cortisol in the unspiked fecal extract and the amount expected corresponding to the amount of standard cortisol added.

As detailed in Carlsson et al. (2016), GCs were extracted from 0.5 g of each fecal sample by rotating overnight (16–18 h) at room temperature in 5 mL of 80% methanol-distilled water. Samples were then centrifuged for 10 min at 2,400*g* and the supernatant (fecal extract) was decanted and stored in glass vials at −20°C until further analysis [25].

Samples were removed from the freezer and thawed at room temperature prior to analysis. For cortisol analysis, 40 μl of fecal extract was evaporated in a fume hood at room temperature and the dried extracts were then reconstituted in 160 μl of assay buffer for a 1:4 dilution. For corticosterone analysis, 80 μl of fecal extract was evaporated in a fume hood at room temperature and the dried extracts were then reconstituted in 160 μl of assay buffer for a 1:2 dilution. Fecal cortisol and corticosterone metabolites were measured using the appropriate EIA protocols described by Majchrzak et al. 2015 and Baxter-Gilbert et al. 2014, respectively [26, 27]. Cortisol antibody and cortisol horseradish peroxidase conjugate dilutions were 1:10,250 and 1:33,400, respectively. Corticosterone antibody and corticosterone-HRP conjugate dilutions were 1:298,000 and 1:100,000, respectively. The detection limits of these assays were 34.5 pg/ml (cortisol) and 82.1 pg/ml (corticosterone). The cross-reactivities of the cortisol and corticosterone EIAs used were 100% to the parent hormone and < 10 or < 15% with other GCs, respectively (C. Munro, University of California, Davis, CA, USA; S1 File).

All concentrations were assayed in duplicate, with the mean of the two results presented as data. Only the duplicates with coefficients of variation (CVs, calculated as (standard deviation/mean) × 100) < 10% were accepted, and if CV was ≥ 10%, duplicate quantitation was repeated on a second run. Data are presented as nanograms of hormone metabolites per gram of wet feces (ng/g). Due to the use of both a cortisol- and a corticosterone-specific EIA for GC metabolite detection in the fecal extracts, the terms “fecal cortisol” and “fecal corticosterone” will be used for simplicity in the results to refer to the FGMs detected by the cortisol and corticosterone EIAs, respectively. Results are presented as descriptive data and plots were done using the R software version 3.4.4 [28].

## 3. Results

### 3.1 EIA validation

Serial dilutions of a pooled muskox fecal extract showed parallel displacement with the standard curves for both the cortisol (Pearson’s correlation coefficient (r) = 0.987, p < 0.01) and corticosterone (r = 0.971, p < 0.01) EIAs (S1 File). Recovery of exogenous hormone added to a pooled muskox fecal extract was 117.6 ± 2.4% (r = 0.999, p < 0.001) for cortisol and 75.5 ± 2.8% (r = 0.999, p < 0.001) for corticosterone (S1 File). Intra-assay CVs were 6.0 and 9.5%, and inter-assay CVs were 9.2 and 3.7%, for the cortisol and corticosterone assays, respectively.

### 3.2 FGMs

Fecal cortisol levels varied substantially within and among individuals, and none of the animals in either challenge showed an increase in fecal cortisol following the injection of ACTH. Results from the cortisol EIA are consequently presented in the S2 File.

**Fig 1:**
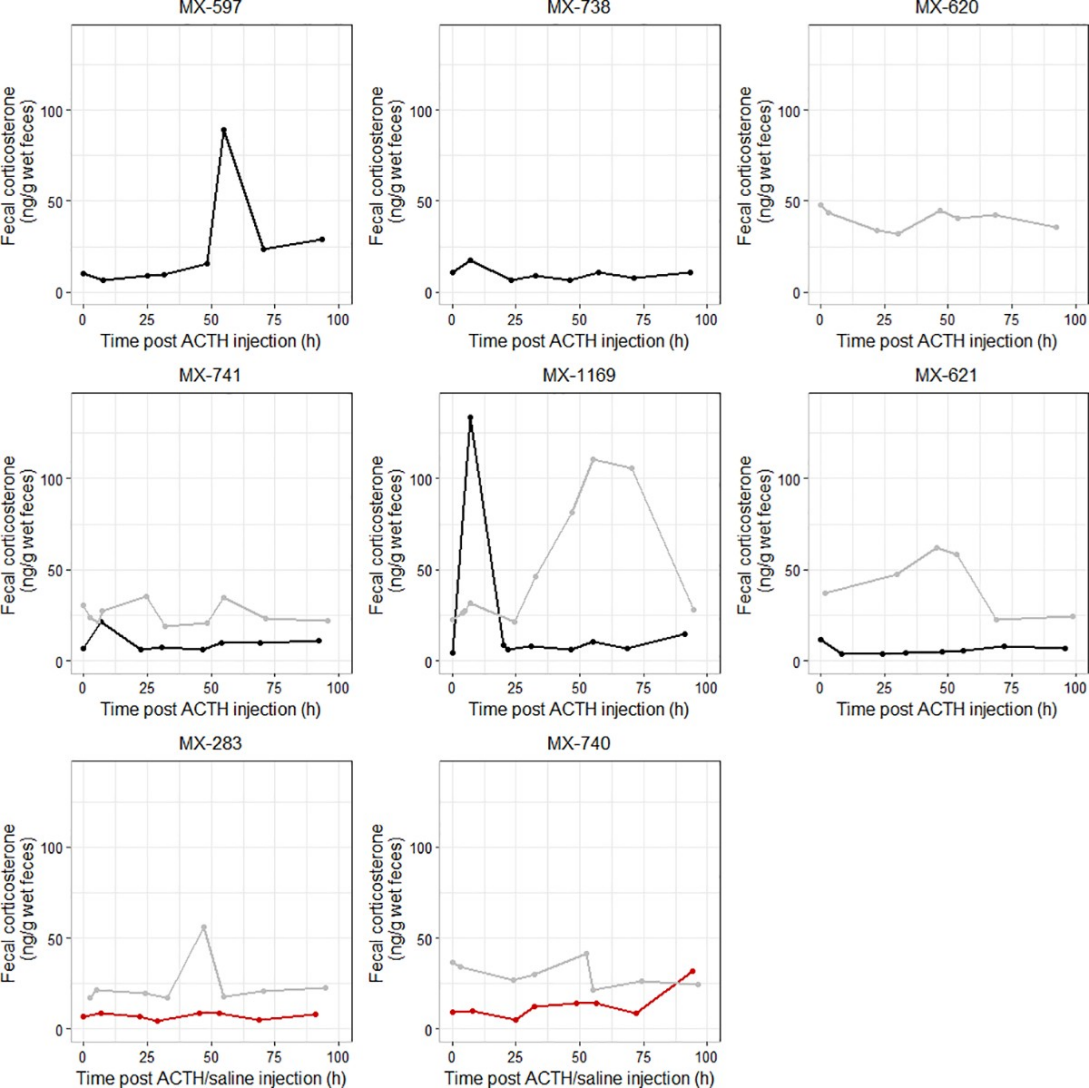
Individual fecal corticosterone levels of the muskoxen as a function of the time following a single injection of ACTH during the winter (ACTH dose 1 IU/kg–n = 6) and/or of ACTH or saline (control) during the summer (ACTH dose 2 IU/kg–n = 5 ACTH-injected and n = 2 controls). Winter data are indicated as grey lines. Data for the ACTH-injected and control animals during the summer challenge correspond to the black and red lines, respectively.

Fecal corticosterone levels varied among individuals during both the winter and summer challenges. Two of six (winter) and two of five (summer) muskoxen showed a clear response following the injection of ACTH (Fig 1 and Table 2). Increases in fecal corticosterone post-ACTH injection occurred earlier and were of shorter duration in the summer than in the winter (Fig 1 and Table 2). Fecal corticosterone levels were also, in general, lower during the summer than during the winter challenge (Fig 1).

**Table 2:** Maximal percentage increase in fecal corticosterone as compared to time 0 levels for the muskoxen given a single injection of ACTH (1 IU/kg) during the winter and/or a single injection of ACTH (2 IU/kg) or saline (control) during the summer, and the respective times post-injection at which it was observed.

	Winter challenge			Summer challenge		
Animal ID	Experimental group	Maximal percentage increase in fecal corticosterone (%)	Time post-injection (h)	Experimental group	Maximal percentage increase in fecal corticosterone (%)	Time post- injection (h)
MX-738	/	/	/	ACTH	64	7
MX-740	ACTH	13	53	Control	237	94
MX-741	ACTH	17	25	ACTH	228	7
MX-620	ACTH	0*	0*	/	/	/
MX-621	ACTH	69	45	ACTH	0*	0*
MX-597	/	/	/	ACTH	776	55
MX-283	ACTH	229	47	Control	25	45
MX-1169	ACTH	394	55	ACTH	2,814	7

*The maximal fecal corticosterone concentration was measured at time 0.

During the winter challenge, MX-283 exhibited a maximal percentage increase in fecal corticosterone of 229% at 47 h post-ACTH injection and levels subsequently decreased by 55 h. For MX-1169, fecal corticosterone concentrations started increasing after 25 h before reaching a peak at 55 h, corresponding to a 394% increase, and decreasing back to levels similar to those measured at time 0 by 95 h. The other four muskoxen (MX-740, MX-741, MX-620, and MX-621) had maximal percentage increases less than 70% (Fig 1 and Table 2).

During the summer challenge, for MX-741 and MX-1169, fecal corticosterone concentrations increased by 228 and 2,814%, respectively, at approximately 7 h post-ACTH injection before decreasing back to levels similar to those measured at time 0 by 22 h. MX-597 exhibited a 776% increase in fecal corticosterone at 55 h. The other two ACTH-injected animals (MX-738 and MX-621) had maximal percentage increases lower than 70% (Fig 1 and Table 2). The two control animals, MX-740 and MX-283, exhibited a maximal percentage increase of 237% at 94 h and 25% at 45 h, respectively (Fig 1 and Table 2).

## 4. Discussion

In this study, we demonstrated that a corticosterone EIA could detect changes in FGM levels following pharmacological stimulation of the HPA axis with the administration of ACTH. By contrast none of the animals in either challenge showed an increase in fecal cortisol in response to the pharmacological challenge. This is likely due to the cortisol antibody not cross-reacting with the metabolites present in the feces of muskoxen. The cortisol EIA will not be discussed further and FGMs in the remainder of this manuscript refer to those measured with the corticosterone EIA. We observed seasonal differences in the timing and duration of the increase in FGMs post-ACTH injection, as well as in FGM levels in general. Despite limitations associated with working with a large non-domesticated arctic ungulate in captivity, this work advances our understanding of FGMs in muskoxen, and provides insights into the interpretation of FGM levels for other species.

During both the winter and summer challenges, there was a high variability among individuals in the presence and magnitude of their response to a single dose of ACTH, as well as in the timing of the peak and duration of the increase in FGMs post-injection. Similar inter-individual variations have been reported in other ungulates (e.g., Rocky mountain goats (*Oreamnos americanus*) [29], reindeer (*Rangifer tarandus tarandus*) [30]; S1 Table). These could be due to a variety of factors, including sex, age, reproductive status, health condition, and/or “individual” characteristics, such as genetic background or past and recent experiences, which may influence GC levels, the responsiveness of the HPA axis, and the metabolism and excretion of FGMs [19, 31]. In this study, the same individual (i.e., MX-1169) exhibited the highest increase in both challenges. Additionally, differences in the age and sex of the muskoxen may explain some of the inter-individual variability in FGM responses. During the summer challenge, MX-1169 (2,814% increase) was a sexually mature female, whereas MX-738 and MX-741, which exhibited peaks of lower magnitude (64 and 228% increase, respectively), were male yearlings. A lower responsiveness of males was found during a pharmacological challenge in reindeer [21] and increased HPA axis responsiveness with age has been described in other vertebrate species [31]. By contrast, reproductive status is likely not an influential factor in this study as (i) we included only non-pregnant females; (ii) breeding occurs in September at this location; and (iii) we did not use sexually mature males in July, a time at which they would have started to exhibit increased agonistic interactions associated with the approach of the rut. Multiple aspects of the social environment, such as the social status of the animals and the stability of the social hierarchy, may also affect GC levels [32]. Even though the dominance hierarchies were well established in the various groups of muskoxen, it is possible that the social interactions and status of individuals influenced our results. Additionally, animal ‘origin’ may have contributed to the inter-individual differences in FGM responses as all except two of the muskoxen were born and raised in captivity, with MX-1169 and MX-283 brought into captivity from the wild as calves. However, this is unlikely, as a study in bighorn sheep (*Ovis canadensis*) found that, once acclimated, animals born in the wild and brought into captivity had similar HPA axis responses to animals born and raised in captivity [33]. A larger sample size would have allowed us to carry out statistical analyses and better assess the influence of sex, age, and other influential factors on the FGM results.

The fecal sampling regime may also have contributed to the inter-individual variations observed. If collections are not sufficiently frequent, the peak response for some animals may be missed. For example, by not collecting the fecal samples voided between 8 and 24 h post-injection during the summer challenge, we likely detected only the beginning of the FGM increase in MX-738 and MX-741 and probably missed the response entirely for MX-597 and MX-621. The possibility of having missed peak samples in some animals emphasizes the importance of collecting and testing, when feasible, feces from all fecal voidance events during the expected time of response. We observed relatively low maximal percentage increases of 13% at 53 h, 17% at 25 h, and 69% at 45h in three animals (MX-740, MX-741, and MX-621, respectively) during the winter challenge (Fig 1), and two other animals (MX-620 –winter and MX-621 –summer) had their maximal FGM levels measured at time 0. These zero and low maximal increases may reflect the unusually high FGM concentrations measured at time 0. We are unaware of the occurrence of a previous stressful event that may have caused these high time 0 levels. Ideally, because of intra-individual variations, multiple samples (rather than just a single sample, as was done in this study) should be collected prior to adrenal stimulation to establish an FGM baseline [19]. Finally, it is possible that some animals exhibited a low or an absence of response to the ACTH administered as has been highlighted in other studies (e.g., [21, 29, 34]).

The timing and magnitude of the peaks in FGMs that we detected, as well as the timing of return to baseline levels, are comparable to those measured following pharmacological challenges in other even-toed ungulate species (S1 Table). The 2,814% increase observed in MX-1169 during the summer challenge sits at the high end of the ranges of FGM peak magnitudes. The summer FGM peak timing of 7 h with a return to time 0 levels by 22 h post-injection would be closest to the responses measured in reindeer [30], caribou (*Rangifer tarandus granti*) [21], cattle (*Bos taurus*) [10, 35], and sheep (*Ovis aries*) [35]. The later peaks and long responses observed during the winter challenge (i.e., peak in FGMs at 47 h with a decrease by 55 h in MX-283 and increase after 25 h followed by a return to time 0 levels by 95 h in MX-1189) are at the high end of the ranges measured in other even-toed ungulate species, but such responses have been observed in reindeer [21] and dromedary camels (*Camelus dromedaries*) [36].

Increases in FGMs post-ACTH injection occurred earlier and were of shorter duration in the summer than in the winter. While the differences observed between the two challenges may be the result of the different doses of ACTH administered (1 IU/kg in the winter and 2 IU/kg in the summer), this is unlikely. In cattle, an increased dosage of ACTH did not affect the peak value or the timing of the cortisol response in serum, but was associated with an elongation of the response [37]. We would, therefore, have expected a longer FGM response in the summer with the higher ACTH dose. While comparisons between the two challenges must be made with caution because of differences in ACTH dosage, sampling times, and animals used (i.e., with a possible influence of the multiple factors discussed for inter-individual variations), our data suggest that metabolism of GCs and intestinal transit time were faster in the summer than in the winter (Fig 1). The few studies that have compared seasonal responses of FGMs to ACTH administration have had differing results depending on the species. A study of reindeer in Norway in which FGMs were measured following a pharmacological challenge in the winter and a stressful event (i.e., calf marking) in the summer found no major differences in the timing of the FGM elevation [30]. A study in white-tailed deer (*Odocoileus virginianus*) detected earlier peaks in FGMs following a pharmacological challenge in the winter (10–13 h post-ACTH injection) compared to the fall (20–24 h) [38]. Finally, a study in cattle found longer lag times between elevated plasma GC levels and peak FGM concentrations following ACTH challenges in the autumn (14.8 ± 0.47 h (mean ± SD)) compared to the spring (8.61 ± 0.26 h) [10]. Seasonal differences in FGM responses may be related to seasonal variations in type and quantity of food intake, as well as differences in liver metabolism and conjugation rates, bacteria action as they further metabolize GCs in the intestines, and fecal output and moisture content [10, 38]. Muskoxen live in a highly seasonal environment characterized by a short summer with access to abundant forage of high quality, during which they accumulate important fat reserves, followed by a long winter of restricted access to generally limited and low-quality forage [3]. To conserve energy in the extreme winter conditions, muskoxen down-regulate their metabolism and lower their energy expenditure and body temperature [39–41], which would explain a possible slower metabolism of GCs during this period. Studies on intestinal transit times in muskoxen have consistently found that these were slowest during the winter (S2 Table) [42–44]. Based on the high seasonal, inter and intra-individual variations highlighted in these studies (S2 Table), we cannot exclude the range of 7-hour to 47-hour intestinal transit time that we detected depending on the season and based on the timing of the FGM peak. Finally, voluntary food intake [45] and frequency of defecation (Di Francesco, personal observation) were higher during the summer, which may have also contributed to the seasonal differences we observed. Radiometabolism studies, which involve the injection of a radiolabeled steroid hormone and subsequent collection of all the excreta voided, typically represent the gold-standard to determine the metabolism and excretion pathways of steroid hormones [19]. Undertaking such a study in muskoxen both in winter and summer would help to refine the seasonal variations in this species.

FGM levels were also, in general, higher during the winter than during the summer challenge (Fig 1). This finding is in line with the lower hair cortisol concentrations measured in the summer than during the fall and winter in wild muskoxen [46]. Similar seasonal variations in FGM levels have been observed in goral (*Naemorhedus griseus*) [47] and red deer (*Cervus elaphus*) [48]. These may reflect the reduced metabolic rate and voluntary food intake of muskoxen in winter with a shift towards catabolic metabolism [48, 49].

Two muskoxen in the summer challenge had significant increases in FGMs at 55h (776%) and 94 h (237%) post-ACTH and saline injection, respectively. It is likely that these correspond to an independent stimulus, rather than a delayed experimental response. While we were not able to identify a possible stressful event that could explain these increases, this may support the corticosterone EIA’s potential to detect biological HPA axis responses.

The performance of the cortisol and corticosterone EIAs used in this study differed. While the corticosterone EIA detected responses to the pharmacological challenge in several muskoxen, the cortisol EIA did not detect any changes post-ACTH injection (S2 File) and was thus not used in subsequent studies. Multiple studies have highlighted differences in assay performance for FGM quantification depending on the species. Due to species-specific differences in hormone metabolism during transit through the gut, the excreted metabolites vary in structure and proportion, and consequently, will only be effectively detected using an antibody that cross-reacts with the specific structures present (S1 Table). For example, a study in giraffes (*Giraffa camelopardalis*) found variations both between the animals and among the six EIAs used to quantify FGMs following an ACTH injection [50], and the contrasting results of two pharmacological challenges done in reindeer may have been due to the use of different EIAs (see S1 Table for study details) [21, 30]. While the corticosterone EIA detected responses in several muskoxen, an EIA more specifically targeting the metabolites excreted by this species may have allowed us to more reliably detect changes in FGMs with greater consistency across animals. Access to high-performance liquid chromatography data would have enabled us to separate and characterize the metabolites excreted by the muskoxen, and consequently to determine whether another assay would have been better suited to measure FGMs in this species.

## 5. Conclusion

We have illustrated, through two pharmacological challenges in captive muskoxen, that the FGM response following the administration of ACTH can be measured in this unique arctic ungulate species using a corticosterone EIA. This is encouraging with respect to using FGMs as a biomarker of HPA axis activity in muskoxen. Fecal GC metabolites have been widely used to inform wildlife conservation in a variety of species and settings (e.g., [51–53]). This tool can now be applied to assess the impact of various stressors (e.g., weather conditions, anthropogenic activities) in muskoxen and to investigate the relationship between FGM levels and health indicators (e.g., parasite richness and infestation intensity or exposure to various bacterial and viral pathogens). This is particularly important to evaluate in the Arctic, where rapid climate warming is leading to increased environmental changes and altered host-pathogen interactions, as evidenced, for example, by the recent range expansion of two major muskox lungworms in the Canadian Arctic Archipelago [54, 55].

Our results also suggest that there are seasonal variations in the metabolism and excretion of GCs, as well as in intestinal transit time, in muskoxen. These findings advance our understanding of the physiological adaptations of mammals living in highly seasonal and extreme environments such as the Arctic, and emphasize the importance of considering seasonality in other species when interpreting FGM levels.

## Supporting information

S1 Table Summary of pharmacological challenges done in other wild and domestic even-toed ungulate species to validate the use of fecal glucocorticoid metabolite levels as a biomarker of hypothalamic-pituitary-adrenal axis activity. NS indicates non-specified information.
(PDF)

S2 Table Summary of the studies measuring intestinal transit times in muskoxen. (PDF)

S1 File Results from the analytical validations of the cortisol and corticosterone enzyme immunoassays. (PDF)

S2 File Fecal cortisol results. (PDF)

S3 File Data from the two ACTH challenges. (XLSX)

## References

[1] Kutz S, Rowell J, Adamczewski J, Gunn A, Cuyler C, Aleuy OA, et al. Muskox Health Ecology Symposium 2016: Gathering to share knowledge on *Umingmak* in a time of rapid change. ARCTIC. 2017;70: 225. 10.14430/arctic4656

[2] Cuyler C, Rowell J, Adamczewski J, Anderson M, Blake J, Bretten T, et al. Muskox status, recent variation, and uncertain future. Ambio. 2019;49: 805–819. 10.1007/s13280-019-01205-x 31187429 PMC6989413

[3] Gunn A, Adamczewski J. Muskox (*Ovibos moschatus*). 2nd ed. Wild Mammals of North America: Biology, Management, and Conservation. 2nd ed. Baltimore, USA and London, UK: The John Hopkins University Press; 2003. pp. 1076–1094.

[4] AMAP. Snow, water, ice and permafrost in the Arctic (SWIPA) 2017. Arctic Monitoring and Assessment Programme (AMAP), Oslo, Norway; 2017. Available: www.amap.no/swipa2017

[5] Kutz SJ, Hoberg EP, Molnár PK, Dobson A, Verocai GG. A walk on the tundra: Host–parasite interactions in an extreme environment. Int J Parasitol Parasites Wildl. 2014;3: 198–208. 10.1016/j.ijppaw.2014.01.002 25180164 PMC4145143

[6] Prewer E, Kutz S, Leclerc LM, Kyle CJ. Already at the bottom? Demographic declines are unlikely further to undermine genetic diversity of a large Arctic ungulate: muskox, *Ovibos moschatus* (Artiodactyla: Bovidae). Biol J Linn Soc. 2020;129: 459–469. 10.1093/biolinnean/blz175

[7] Romero LM, Butler LK. Endocrinology of stress. Int J Comp Psychol. 2007;20: 89–95.

[8] Koren L, Whiteside D, Fahlman Å, Ruckstuhl K, Kutz S, Checkley S, et al. Cortisol and corticosterone independence in cortisol-dominant wildlife. Gen Comp Endocrinol. 2012;177: 113–119. 10.1016/j.ygcen.2012.02.020 22449618

[9] Möstl E, Palme R. Hormones as indicators of stress. Domest Anim Endocrinol. 2002;23: 67–74. 10.1016/s0739-7240(02)00146-7 12142227

[10] Morrow CJ, Kolver ES, Verkerk GA, Matthews LR. Fecal glucocorticoid metabolites as a measure of adrenal activity in dairy cattle. Gen Comp Endocrinol. 2002;126: 229–241. 10.1006/gcen.2002.7797 12030779

[11] Palme R, Fischer P, Schildorfer H, Ismail MN. Excretion of infused 14C-steroid hormones via faeces and urine in domestic livestock. Anim Reprod Sci. 1996;43: 43–63.

[12] Touma C, Palme R. Measuring fecal glucocorticoid metabolites in mammals and birds: The importance of validation. Ann N Y Acad Sci. 2005;1046: 54–74. 10.1196/annals.1343.006 16055843

[13] Wasser SK, Hunt KE, Brown JL, Cooper K, Crockett CM, Bechert U, et al. A generalized fecal glucocorticoid assay for use in a diverse array of nondomestic mammalian and avian species. Gen Comp Endocrinol. 2000;120: 260–275. 10.1006/gcen.2000.7557 11121291

[14] Palme R, Rettenbacher S, Touma C, El-Bahr SM, Möstl E. Stress hormones in mammals and birds: Comparative aspects regarding metabolism, excretion, and noninvasive measurement in fecal samples. Ann N Y Acad Sci. 2005;1040: 162–171. 10.1196/annals.1327.021 15891021

[15] Sheriff MJ, Dantzer B, Delehanty B, Palme R, Boonstra R. Measuring stress in wildlife: Techniques for quantifying glucocorticoids. Oecologia. 2011;166: 869–887. 10.1007/s00442-011-1943-y 21344254

[16] Möstl E, Maggs JL, Schrötter G, Besenfelder U, Palme R. Measurement of cortisol metabolites in faeces of ruminants. Vet Res Commun. 2002;26: 127–139. 10.1023/a:1014095618125 11922482

[17] Möstl E, Messmann S, Bagu E, Robia C, Palme R. Measurement of glucocorticoid metabolite concentrations in faeces of domestic livestock. Transbound Emerg Dis. 1999;46: 621–631. 10.1046/j.1439-0442.1999.00256.x 10638300

[18] Möstl E, Rettenbacher S, Palme R. Measurement of corticosterone metabolites in birds’ droppings: An analytical approach. Ann N Y Acad Sci. 2005;1046: 17–34. 10.1196/annals.1343.004 16055841

[19] Palme R. Non-invasive measurement of glucocorticoids: Advances and problems. Physiol Behav. 2019;199: 229–243. 10.1016/j.physbeh.2018.11.021 30468744

[20] Di Francesco J, Mastromonaco GF, Checkley SL, Blake J, Rowell JE, Kutz S. Qiviut cortisol reflects hypothalamic–pituitary–adrenal axis activity in muskoxen (*Ovibos moschatus*). Gen Comp Endocrinol. 2021;306: 113737. 10.1016/j.ygcen.2021.113737 33610573

[21] Ashley NT, Barboza PS, Macbeth BJ, Janz DM, Cattet MRL, Booth RK, et al. Glucocorticosteroid concentrations in feces and hair of captive caribou and reindeer following adrenocorticotropic hormone challenge. Gen Comp Endocrinol. 2011;172: 382–391. 10.1016/j.ygcen.2011.03.029 21501613

[22] Dehnhard M, Clauss M, Lechner-Doll M, Meyer HHD, Palme R. Noninvasive monitoring of adrenocortical activity in roe deer (*Capreolus capreolus*) by measurement of fecal cortisol metabolites. Gen Comp Endocrinol. 2001;123: 111–120. 10.1006/gcen.2001.7656 11551112

[23] Ganswindt A, Tordiffe ASW, Stam E, Howitt MJ, Jori F. Determining adrenocortical activity as a measure of stress in African buffalo (*Syncerus caffer*) based on faecal analysis. Afr Zool. 2012;47: 262–269.

[24] U.S. Climate Data. [cited 23 Dec 2020]. Available: https://www.usclimatedata.com/climate/fairbanks/alaska/united-states/usak0083

[25] Carlsson AM, Mastromonaco G, Vandervalk E, Kutz S. Parasites, stress and reindeer: Infection with abomasal nematodes is not associated with elevated glucocorticoid levels in hair or faeces. Conserv Physiol. 2016;4: cow058. 10.1093/conphys/cow058 27957334 PMC5147723

[26] Baxter-Gilbert JH, Riley JL, Mastromonaco GF, Litzgus JD, Lesbarreres D. A novel technique to measure chronic levels of corticosterone in turtles living around a major roadway. Conserv Physiol. 2014;2: cou036. 10.1093/conphys/cou036 27293657 PMC4806746

[27] Majchrzak YN, Mastromonaco GF, Korver W, Burness G. Use of salivary cortisol to evaluate the influence of rides in dromedary camels. Gen Comp Endocrinol. 2015;211: 123–130. 10.1016/j.ygcen.2014.11.007 25452030

[28] R Core Team. R: a Language and Environment for Statistical Computing. R Foundation for Statistical Computing, Vienna, Austria.; 2019. Available: https://www.R-project.org/

[29] Dulude-de Broin F, Côté SD, Whiteside DP, Mastromonaco GF. Faecal metabolites and hair cortisol as biological markers of HPA-axis activity in the Rocky mountain goat. Gen Comp Endocrinol. 2019;280: 147–157. 10.1016/j.ygcen.2019.04.022 31009603

[30] Özkan Gülzari Ş, Jørgensen GHM, Eilertsen SM, Hansen I, Hagen SB, Fløystad I, et al. Measuring faecal glucocorticoid metabolites to assess adrenocortical activity in reindeer. Animals. 2019;9: 987. 10.3390/ani9110987 31752137 PMC6912703

[31] Dantzer B, Fletcher QE, Boonstra R, Sheriff MJ. Measures of physiological stress: A transparent or opaque window into the status, management and conservation of species? Conserv Physiol. 2014;2: cou023. 10.1093/conphys/cou023 27293644 PMC4732472

[32] Creel S, Dantzer B, Goymann W, Rubenstein DR. The ecology of stress: Effects of the social environment. Boonstra R, editor. Funct Ecol. 2013;27: 66–80. 10.1111/j.1365-2435.2012.02029.x

[33] Coburn S, Salman M, Rhyan J, Keefe T, McCollum M, Aune K, et al. Comparison of endocrine response to stress between captive-raised and wild-caught bighorn sheep. J Wildl Manag. 2010;74: 532–538. 10.2193/2008-152

[34] Mastromonaco GF, Gunn K, McCurdy-Adams H, Edwards DB, Schulte-Hostedde AI. Validation and use of hair cortisol as a measure of chronic stress in eastern chipmunks (*Tamias striatus*). Conserv Physiol. 2014;2: cou055. 10.1093/conphys/cou055 27293676 PMC4732495

[35] Palme R, Robia C, Messmann S, Hofer J, Möstl E. Measure of faecal cortisol metabolites in ruminants: A non-invasive parameter for adrenal function. Wien Tierärztl Monatsschrift. 1999;86: 237–241.

[36] Sid-Ahmed O-E, Sanhouri A, Elwaseela B-E, Fadllalah I, Mohammed G-EE, Möstl E. Assessment of adrenocortical activity by non-invasive measurement of faecal cortisol metabolites in dromedary camels (*Camelus dromedarius*). Trop Anim Health Prod. 2013;45: 1453–1458. 10.1007/s11250-013-0374-7 23430659

[37] Lay DC, Friend TH, Randel RD, Jenkins OC, Neuendorff DA, Kapp GM, et al. Adrenocorticotropic hormone dose response and some physiological effects of transportation on pregnant Brahman cattle. J Anim Sci. 1996;74: 1806–1811. 10.2527/1996.7481806x 8856435

[38] Millspaugh JJ, Washburn BE, Milanick MA. Non-invasive techniques for stress assessment in white-tailed deer. Wildl Soc Bull. 2002;30: 899–907.

[39] Adamczewski J, Flood PF. Seasonal patterns in body composition and reproduction of female muskoxen (*Ovibos moschatus*). J Zool. 1997;241: 245–269.

[40] Lawler JP, White RG. Seasonal changes in metabolic rates in muskoxen following twenty- four hours of starvation. Rangifer. 1997;17: 135. 10.7557/2.17.3.1365

[41] Schmidt NM, Grøndahl C, Evans AL, Desforges J-P, Blake J, Hansen LH, et al. On the interplay between hypothermia and reproduction in a high arctic ungulate. Sci Rep. 2020;10: 1514. 10.1038/s41598-020-58298-8 32001737 PMC6992616

[42] Adamczewski JZ, Flood PF, Chaplin RK, Schaefer JA. Seasonal variation in intake and digestion of a high-roughage diet by muskoxen. Can J Anim Sci. 1994;74: 305–313. 10.4141/cjas94-042

[43] Barboza PS, Peltier TC, Forster RJ. Ruminal fermentation and fill change with season in an Arctic grazer: Responses to hyperphagia and hypophagia in muskoxen (*Ovibos moschatus*). Physiol Biochem Zool. 2006;79: 497–513. 10.1086/501058 16691516

[44] Holleman DF, White RG, Frisby K, Jourdan M, Henrichsen P, Tallas PG. Food passage rates in captive muskoxen as measured with non-absorbed radiolabeled markers. Biol Pap Univ Alsk Spec Rep. 1984; 188–192.

[45] White RG, Holleman DF, Wheat P, Tallas PG, Jourdan M. Seasonal changes in voluntary intake and digestibility of diets by captive muskoxen. Biol Pap Univ Alsk Spec Rep. 1984; 193–194.

[46] Di Francesco J, Navarro-Gonzalez N, Wynne-Edwards K, Peacock S, Leclerc L-M, Tomaselli M, et al. Qiviut cortisol in muskoxen as a potential tool for informing conservation strategies. Conserv Physiol. 2017;5: cox052. 10.1093/conphys/cox052 28948023 PMC5601961

[47] Khonmee J, Brown JL, Rojanasthien S, Aunsusin A, Thumasanukul D, Kongphoemphun A, et al. Gender, season and management affect fecal glucocorticoid metabolite concentrations in captive goral (*Naemorhedus griseus*) in Thailand. PLOS ONE. 2014;9: e91633. 10.1371/journal.pone.0091633 24637886 PMC3956719

[48] Huber S, Palme R, Arnold W. Effects of season, sex, and sample collection on concentrations of fecal cortisol metabolites in red deer (*Cervus elaphus*). Gen Comp Endocrinol. 2003;130: 48–54. 10.1016/s0016-6480(02)00535-x 12535624

[49] Goymann W. On the use of non‐invasive hormone research in uncontrolled, natural environments the problem with sex, diet, metabolic rate and the individual. Methods Ecol Evol. 2012;3: 757–765.

[50] Bashaw MJ, Sicks F, Palme R, Schwarzenberger F, Tordiffe ASW, Ganswindt A. Non-invasive assessment of adrenocortical activity as a measure of stress in giraffe (*Giraffa camelopardalis*). BMC Vet Res. 2016;12. 10.1186/s12917-016-0641-8 27756312 PMC5070010

[51] Atwood MP, Kie JG, Millspaugh JJ, Matocq MD, Bowyer RT. Condition of mule deer during winter: stress and spatial overlap with North American elk. Mammal Res. 2020;65: 349–358. 10.1007/s13364-019-00474-x

[52] Zbyryt A, Bubnicki JW, Kuijper DPJ, Dehnhard M, Churski M, Schmidt K. Do wild ungulates experience higher stress with humans than with large carnivores? Wong B, editor. Behav Ecol. 2018;29: 19–30. 10.1093/beheco/arx142

[53] Zwijacz-Kozica T, Selva N, Barja I, Silván G, Martínez-Fernández L, Illera JC, et al. Concentration of fecal cortisol metabolites in chamois in relation to tourist pressure in Tatra National Park (South Poland). Acta Theriol (Warsz). 2013;58: 215–222. 10.1007/s13364-012-0108-7

[54] Kutz S, Checkley S, Verocai GG, Dumond M, Hoberg EP, Peacock R, et al. Invasion, establishment, and range expansion of two parasitic nematodes in the Canadian Arctic. Glob Change Biol. 2013;19: 3254–3262. 10.1111/gcb.12315 23828740

[55] Kafle P, Peller P, Massolo A, Hoberg E, Leclerc L-M, Tomaselli M, et al. Range expansion of muskox lungworms track rapid arctic warming: Implications for geographic colonization under climate forcing. Sci Rep. 2020;10: 17323. 10.1038/s41598-020-74358-5 33057173 PMC7560617

