# Supplementary material for: Fecal glucocorticoid metabolites reflect hypothalamic–pituitary–adrenal axis activity in muskoxen (Ovibos moschatus)
Source: PLoS One. 2021 Apr 14;16(4):e0249281. doi: 10.1371/journal.pone.0249281 (PMC8046187; doi:10.1371/journal.pone.0249281)
Supplement: S1 Table — NS indicates non-specified information. (PDF) [file pone.0249281.s001.pdf]

<u>S1 Table</u>: Summary of pharmacological challenges done in other wild and domestic even-toed ungulate species to validate the use of fecal glucocorticoid metabolite levels as a biomarker of hypothalamic-pituitary-adrenal axis activity. NS indicates non-specified information.

**Species**	**Sample size**	**ACTH formulation (F), dosage (D), and route of administration (R)⁺**	**Timing of FGM peak (h post-injection – range unless specified otherwise)**	**Magnitude of FGM peak (% increase from baseline – range unless specified otherwise)**	**Timing of return to baseline (h post-injection)**	**Assay****	**Reference**
**Wild species**							
African buffalo (*Syncerus caffer*)	1 ♂ yearling and 1 adult ♀	F: Synacthen Depot, Hoffman-La Roche AG<br>D: 150 IU total<br>R: IM	♂ = 8-22 h (11,17-DOA) and 22 h (3α,11oxo-CM)<br>♀ = 7-21 h (both EIAs)	♂ = 1,500% (11,17-DOA) and 1,200% (3α,11oxo-CM);<br>♀ = 1,000% (11,17-DOA) and 900% (3α,11oxo-CM)	♂ = 48 h (11,17-DOA)<br>♀ = 29 h (11,17-DOA)	11,17-DOA EIA and 3α,11oxo-CM EIA	Ganswindt et al., 2012
African buffalo (*Syncerus caffer*)	3 adult ♂ and 5 adult ♀	F: Synacthen Depot, Novartis<br>D: 1 IU/kg<br>R: IM	RIA: average = 20 h (95% CI = 13-27 h)<br>EIA: average = 23 h (95% CI = 15-30 h)	RIA: average = 371%<br>EIA: average = 1,247%<br>Both are expressed after accounting for week, age, sex, and buffalo ID	NS (> 50 h for RIA and EIA*)	Corticosterone RIA (MP Biomedicals) and cortisol EIA (Enzo Life Sciences Inc.)	Spaan et al., 2017
Alpaca (*Vicugna pacos*)	3 intact ♂, 3 castrated ♂, and 3 ♀ all adults	F: Synacthen, Defiante Farmaceutica<br>D: 25 IU total<br>R: IV	Median = 33 h	963% (value NS)	> 48 h (no samples collected past this time)	11,17-DOA EIA	Arias et al., 2013
Brown brocket deer (*Mazama gouazoubira*)	1 adult ♂ and 1 adult ♀	F: Synacthen Depot, Novartis<br>D: 25 IU total<br>R: IM	♂ = 29 h*<br>♀ = 24 h*	♂ = 313%<br>♀ = 334%	♂ = 40 h*<br>♀ = 33 h*	Cortisol EIA (Munro)	Christofoletti et al., 2010
Caribou (*Rangifer tarandus granti*)	5 adult ♂ and 5 adult ♀	F: ACTH gel form, Meds for Vets Inc.<br>D: 2 IU/kg<br>R: IM	♂ = 8 h<br>♀ = 8 h<br>Determined after averaging values of all animals	♂ = 190%<br>♀ = 154%<br>Determined after averaging values of all animals	♂ = 24 h<br>♀ = 48 h	Corticosterone RIA (MP Biomedicals)	Ashley et al., 2011
Collared peccary (*Pecari tajacu*)	6 adult ♂	F: Synacthen Depot, Novartis<br>D: 0.25 IU/kg<br>R: IM	24 h	30-390%	48 h	Cortisol EIA (Munro)	Coradello et al., 2012

Dromedary camel (*Camelus dromedarius*)	2 adult ♂ and 3 adult ♀	F: Synacthen Depot, Novartis<br>D: 50 IU total<br>R: IV	♂ = 24 h<br>♀ = 36-48 h	♂ = 210-734%<br>♀ = 330-702%	♂ = 36 h<br>♀ = 72 h	3α,11oxo-CM EIA	Sid-Ahmed et al., 2013
Fallow deer (*Dama dama*)	3 ♂ yearlings	F: $ACTH_{1-24}$ Sigma-Aldrich<br>D: 0.6 IU/kg<br>R: IM	Deer 1 = 18 h<br>Deer 2 = 22 h<br>Deer 3 = 22 h	Deer 1 = 290%<br>Deer 2 = 430%<br>Deer 3 = 290%	NS (no samples collected past 22 h)	11,17-DOA EIA	Konjević et al., 2011
Giraffe (*Giraffa camelopardalis*)	2 adult ♂	F: Synacthen Depot, Novartis<br>D: 1 IU/kg (giraffe 1) and 0.7 IU/kg (giraffe 2)<br>R: IM	Giraffe 1:<br>13.5-19.5 h (cortisol)<br>50.5-52.5 h (corticosterone)<br>13.5-50.5 h (11,17-DOA)<br>11.5-50.5 h (3α,11oxo-CM)<br>13.5-35.5 h (3β,11β-diol CM)<br>30-35 h (3α,11β-diol CM)<br>Giraffe 2:<br>No peak (cortisol)<br>No peak (corticosterone)<br>20.5-27.5 h (11,17-DOA)<br>24-29 h (3α,11oxo-CM)<br>23.2-28.2 h (3β,11β-diol CM)<br>No peak (3α,11β-diol CM)	Giraffe 1:<br>160% (cortisol)<br>60% (corticosterone)<br>3,240% (11,17-DOA)<br>1720% (3α,11oxo-CM)<br>310% (3β,11β-diol CM)<br>160% (3α,11β-diol CM)<br>Giraffe 2:<br>No peak (cortisol)<br>No peak (corticosterone)<br>860% (11,17-DOA)<br>190% (3α,11oxo-CM)<br>190% (3β,11β-diol CM)<br>No peak (3α,11β-diol CM)	Giraffe 1:<br>21 h (cortisol)<br>53.5 h (corticosterone)<br>51.5 h (11,17-DOA)<br>51.5 h (3α,11oxo-CM)<br>37 h (3β,11β-diol CM)<br>35.5 h (3α,11β-diol CM)<br>Giraffe 2:<br>No peak (cortisol)<br>No peak (corticosterone)<br>44.7 h (11,17-DOA)<br>44.7 h (3α,11oxo-CM)<br>44.7 h (3β,11β-diol CM)<br>No peak (3α,11β-diol CM)	Cortisol EIA (Munro), corticosterone EIA (Munro), 11,17-DOA EIA, 3α,11oxo-CM EIA, 3β,11β-diol CM, and 3α,11β-diol CM	Bashaw et al., 2016
Llama (*Lama glama*)	3 ♀ and 3 ♂ all adults	F: Synacthen, Defiante Farmaceutica<br>D: 25 IU total<br>R: IV	Median = 28 h	805% (value NS)	> 48 h (no samples collected past this time)	11,17-DOA EIA	Arias et al., 2013
Red deer (*Cervus elaphus*)	6 ♀ (age NS)	F: Synacthen, Ciba-Geigy<br>D: 0.5 IU/kg<br>R: IM	Average = 19 h<br>(range* = 16-29 h)	668-2,021%	20-45 h*	3α,11oxo-CM EIA	Huber et al., 2003
Reindeer (*Rangifer tarandus tarandus*)	8 adult ♂	F: Synacthen, CD Pharmaceuticals AB<br>D: 25 IU total<br>R: IM	7 h in 5 individuals and 14, 16, 24 h in 3 other individuals<br>Magnitude:	100-1,580%	Highly variable among individuals (NS)	3α,11oxo-CM EIA	Özkan Gülzari et al., 2019
Reindeer (*Rangifer tarandus tarandus*)	5 ♂ and 5 ♀ all ≈ 1 year	F: ACTH gel form, Meds for Vets Inc.<br>D: 8 IU/kg<br>R: IM	♂ = no response (NS)<br>♀ = 24 h<br>Determined after averaging values of all animals	♂ = no response (NS)<br>♀ = 85%<br>Determined after averaging values of all animals	♂ = no response (NS)<br>♂ > 72 h	Corticosterone RIA (MP Biomedicals)	Ashley et al., 2011

Rocky mountain goat (*Oreamnos americanus*)	1 adult ♂ and 2 adult ♀	F: Synacthen Depot, Novartis<br>D: 50 IU total<br>R: IM	♂ = 32 h<br>♀ n°1 = 21 h<br>♀ n°2 = 20 h	♂ = 192%<br>♀ n°1 = 223%<br>♀ n°2 = 131%	NS in 2 ♀ as not sampled long enough and 40 h in ♂*	Cortisol EIA (Munro)	Dulude-de Broin et al., 2019
Roe deer (*Capreolus capreolus*)	5 castrated ♂ all 9-11 months	F: Synacthen, Ciba-Geigy<br>D: 25 IU total<br>R: IM	6-23 h	504-1,350%	28-31 h	11,17-DOA EIA	Dehnhard et al., 2001
White-tailed deer (*Odocoileus virginianus*)	4 adult ♀ (2 in October and 2 in March)	F: Cortrosyn, Organon Inc.<br>D: 50 IU total<br>R: IM	October: 20-24 h<br>March: 10-13 h	October: 150%<br>March: 240-253%	30-35 h in October and March*	Corticosterone RIA (ICN Pharmaceuticals)	Millspaugh et al., 2002
**Domestic species**							
Cattle (*Bos taurus*)	3 ♀ and 3 ♂ (age NS)	F: Synacthen, Ciba-Geigy<br>D: 100 IU total<br>R: IV	6.0-19 h (after peak in blood cortisol)	230-2,440%	18-44 h	11,17-DOA EIA	Palme et al., 1999
Cattle (*Bos taurus*)	10 adult ♀ (5 in autumn and 5 in spring)	F: Synacthen Depot, Novartis<br>D: 5 IU total twice (at a 2-h interval)<br>R: IV	Autumn: 14-18 h (both EIAs)<br>Spring: 8-10 h (corticosterone)	Autumn: 528% (11,17-DOA) and 196% (corticosterone)<br>Spring: 227% (corticosterone)<br>All values were calculated between the baseline and peak averages	Autumn: 21 h (both EIAs*)<br>Spring: 21 h (corticosterone*)	11,17-DOA EIA and corticosterone RIA (ICN Pharmaceuticals)	Morrow et al., 2002
Goats (*Capra hircus*)	20 adult ♀	F: Synacthen Depot, Novartis<br>D: 50 IU total<br>R: IM	Mean ± SD = 13 ± 1 h (both EIAs)	11,17-DOA: 510-3,160%<br>3α,11oxo-CM: 540-3,050%	NS (≈ 24 h, but > 24 h in some individuals)	11,17-DOA EIA and 3α,11oxo-CM EIA	Kleinsasser et al., 2010
Sheep (*Ovis aries*)	3 ♀ and 3 ♂ (age NS)	F: Synacthen, Ciba-Geigy<br>D: 50 IU total<br>R: IV	6.0-19 h (after peak in blood cortisol)	230-1,510%	18-44 h	11,17-DOA EIA	Palme et al., 1999

[+]IM for intramuscular and IV for intravenous. *Values read from graphs presented in publications – these are approximate values. **The 11,17-DOA EIA is described in Palme and Möstl, (1997), the 3α,11oxo-CM EIA in Möstl et al., (2002), the 3β,11β-diol CM EIA in Touma et al., (2003), and the 3α,11β-diol CM EIA in Ganswindt et al., (2003).

References:

Arias, N., Requena, M., Palme, R., 2013. Measuring faecal glucocorticoid metabolites as a non-invasive tool for monitoring adrenocortical activity in South American camelids. Anim. Welf. 22, 25–31. https://doi.org/10.7120/09627286.22.1.025

Ashley, N.T., Barboza, P.S., Macbeth, B.J., Janz, D.M., Cattet, M.R.L., Booth, R.K., Wasser, S.K., 2011. Glucocorticosteroid concentrations in feces and hair of captive caribou and reindeer following adrenocorticotropic hormone challenge. Gen. Comp. Endocrinol. 172, 382–391. https://doi.org/10.1016/j.ygcen.2011.03.029

Bashaw, M.J., Sicks, F., Palme, R., Schwarzenberger, F., Tordiffe, A.S.W., Ganswindt, A., 2016. Non-invasive assessment of adrenocortical activity as a measure of stress in giraffe (*Giraffa camelopardalis*). BMC Vet. Res. 12. https://doi.org/10.1186/s12917-016-0864-8

Christofoletti, M.D., Pereira, R.J.G., Duarte, J.M.B., 2010. Influence of husbandry systems on physiological stress reactions of captive brown brocket (*Mazama gouazoubira*) and marsh deer (*Blastocerus dichotomus*)—noninvasive analysis of fecal cortisol metabolites. Eur. J. Wildl. Res. 56, 561–568. https://doi.org/10.1007/s10344-009-0350-8

Coradello, M.A., Morais, R.N., Roper, J., Spercoski, K.M., Massuda, T., Nogueira, S.S.C., Nogueira-Filho, S.L.G., 2012. Validation of a fecal glucocorticoid metabolite assay for collared peccaries (*Pecari Tajacu*). J. Zoo Wildl. Med. 43, 275–282. https://doi.org/10.1638/2011-0046.1

Dehnhard, M., Clauss, M., Lechner-Doll, M., Meyer, H.H.D., Palme, R., 2001. Noninvasive Monitoring of Adrenocortical Activity in Roe Deer (*Capreolus capreolus*) by Measurement of Fecal Cortisol Metabolites. Gen. Comp. Endocrinol. 123, 111–120. https://doi.org/10.1006/gcen.2001.7656

Dulude-de Broin, F., Côté, S.D., Whiteside, D.P., Mastromonaco, G.F., 2019. Faecal metabolites and hair cortisol as biological markers of HPA-axis activity in the Rocky mountain goat. Gen. Comp. Endocrinol. 280, 147–157. https://doi.org/10.1016/j.ygcen.2019.04.022

Ganswindt, A., Palme, R., Heistermann, M., Borragan, S., Hodges, J.K., 2003. Non-invasive assessment of adrenocortical function in the male African elephant (*Loxodonta africana*) and its relation to musth. Gen. Comp. Endocrinol. 134, 156–166. https://doi.org/10.1016/S0016-6480(03)00251-X

Ganswindt, A., Tordiffe, A.S.W., Stam, E., Howitt, M.J., Jori, F., 2012. Determining adrenocortical activity as a measure of stress in African buffalo (*Syncerus caffer*) based on faecal analysis. Afr. Zool. 47, 262–269.

Huber, S., Palme, R., Zenker, W., Mostl, E., 2003. Non-Invasive Monitoring of the Adrenocortical Response in Red Deer. J. Wildl. Manag. 67, 258. https://doi.org/10.2307/3802767

Kleinsasser, C., Graml, C., Klobetz-Rassam, E., Barth, K., Waiblinger, S., Palme, R., 2010. Physiological validation of a non-invasive method for measuring adrenocortical activity in goats. Wien. Tierärztl. Monatsschrift 97, 259–262.

Konjević, D., Janicki, Z., Slavica, A., Severin, K., Krapinec, K., Božić, F., Palme, R., 2011. Non-invasive monitoring of adrenocortical activity in free-ranging fallow deer (*Dama dama L.*). Eur. J. Wildl. Res. 57, 77–81. https://doi.org/10.1007/s10344-010-0401-1

Millspaugh, J.J., Washburn, B.E., Milanick, M.A., 2002. Non-Invasive Techniques for Stress Assessment in White-Tailed Deer. Wildl. Soc. Bull. 30, 899–907.

Morrow, C.J., Kolver, E.S., Verkerk, G.A., Matthews, L.R., 2002. Fecal Glucocorticoid Metabolites as a Measure of Adrenal Activity in Dairy Cattle. Gen. Comp. Endocrinol. 126, 229–241. https://doi.org/10.1006/gcen.2002.7797

Möstl, E., Maggs, J.L., Schrötter, G., Besenfelder, U., Palme, R., 2002. Measurement of Cortisol Metabolites in Faeces of Ruminants. Vet. Res. Commun. 26, 127–139.

Özkan Gülzari, Ş., Jørgensen, G.H.M., Eilertsen, S.M., Hansen, I., Hagen, S.B., Fløystad, I., Palme, R., 2019. Measuring Faecal Glucocorticoid Metabolites to Assess Adrenocortical Activity in Reindeer. Animals 9, 987. https://doi.org/10.3390/ani9110987

Palme, R., Möstl, E., 1997. Measurement of cortisol metabolites in faeces of sheep as a parameter of cortisol. Int. J. Mamm. Biol. 62 (Supplement 2), 192–197.

Palme, R., Robia, C., Messmann, S., Hofer, J., Möstl, E., 1999. Measure of faecal cortisol metabolites in ruminants: a non-invasive parameter for adrena function. Wien. Tierärztl. Monatsschrift 86, 237–241.

Sid-Ahmed, O.-E., Sanhouri, A., Elwaseela, B.-E., Fadllalah, I., Mohammed, G.-E.E., Möstl, E., 2013. Assessment of adrenocortical activity by non-invasive measurement of faecal cortisol metabolites in dromedary camels (*Camelus dromedarius*). Trop. Anim. Health Prod. 45, 1453–1458. https://doi.org/10.1007/s11250-013-0374-7

Spaan, J.M., Pitts, N., Buss, P., Beechler, B., Ezenwa, V.O., Jolles, A.E., 2017. Noninvasive measures of stress response in African buffalo (*Syncerus caffer*) reveal an age-dependent stress response to immobilization. J. Mammal. 98, 1288–1300. https://doi.org/10.1093/jmammal/gyx073

Touma, C., Sachser, N., Möstl, E., Palme, R., 2003. Effects of sex and time of day on metabolism and excretion of corticosterone in urine and feces of mice. Gen. Comp. Endocrinol. 130, 267–278. https://doi.org/10.1016/S0016-6480(02)00620-2
