# Supplementary material for: Fecal glucocorticoid metabolites reflect hypothalamic–pituitary–adrenal axis activity in muskoxen (Ovibos moschatus)
Source: PLoS One. 2021 Apr 14;16(4):e0249281. doi: 10.1371/journal.pone.0249281 (PMC8046187; doi:10.1371/journal.pone.0249281)
Supplement: S2 Table — (PDF) [file pone.0249281.s002.pdf]

S2 Table: **Summary of the studies measuring intestinal transit times in muskoxen**.

<table>
<tr><th>Study</th><th>Intestinal retention time (IRT)</th><th>Description and study design</th></tr>
<tr><td>Holleman et al., 1984</td><td>
<table>
<tr><th></th><th>Fluid marker (h)*</th><th>Particulate marker (h)</th></tr>
<tr><td colspan="3">Summer</td></tr>
<tr><td>Pen-fed</td><td>6.5</td><td>7.1</td></tr>
<tr><td>Pasture</td><td>3.2</td><td>4.4</td></tr>
<tr><td colspan="3">Winter</td></tr>
<tr><td>Pen-fed</td><td>9.1</td><td>9.5</td></tr>
<tr><td>Pasture</td><td>4.8</td><td>0.7</td></tr>
</table>
</td><td>Markers (chromium-51 complexed with EDTA (fluid) and ruthenium-103 chloride (particulate)) fed by way of a capsule or by dosing a portion of the food to 3-9 young muskoxen (age and sex not specified)
Calculation of IRT using a compartmental model</td></tr>
<tr><td>Adamczewski et al., 1994</td><td>March: 21.0 ± 1.1 h (mean ± SE)
July: 14.7 ± 0.5 h</td><td>Chromium-mordanted hay (particulate) fed to 6 non-breeding adult ♀
Calculation of IRT using a compartmental model</td></tr>
<tr><td>Barboza et al., 2006</td><td>
<table>
<tr><th></th><th>Fluid marker (h)[+]</th><th>Particulate marker (h)</th></tr>
<tr><td>May-June</td><td>32.1 ± 20.3</td><td>29.4 ± 15.0</td></tr>
<tr><td>Aug-Sept</td><td>28.1 ± 9.1</td><td>26.9 ± 10.3</td></tr>
<tr><td>Feb-March</td><td>33.5 ± 13.9</td><td>36.8 ± 12.1</td></tr>
</table>
</td><td>Cobalt-EDTA (fluid) and ytterbium-fiber (particulate) given in solution and in gel capsules, respectively, at a duodenal cannula to 4-6 castrated adult ♂
Direct measurement of IRT</td></tr>
</table>

*Mean IRTs were calculated using the rumen turnover time (RTT), transit time (TT), and total mean retention time (TMRT) provided in the publication as IRT = TMRT – (RTT + TT). No SD or SE could, however, be calculated without access to the raw data. [+]All results are presented as mean ± SD

References:

Adamczewski, J.Z., Flood, P.F., Chaplin, R.K., Schaefer, J.A., 1994. Seasonal variation in intake and digestion of a high-roughage diet by muskoxen. Can. J. Anim. Sci. 74, 305–313. https://doi.org/10.4141/cjas94-042

Barboza, P.S., Peltier, T.C., Forster, R.J., 2006. Ruminal Fermentation and Fill Change with Season in an Arctic Grazer: Responses to Hyperphagia and Hypophagia in Muskoxen (*Ovibos moschatus*). Physiol. Biochem. Zool. 79, 497–513. https://doi.org/10.1086/501058

Holleman, D.F., White, R.G., Frisby, K., Jourdan, M., Henrichsen, P., Tallas, P.G., 1984. Food passage rates in captive muskoxen as measured with non-absorbed radiolabeled markers. Biol. Pap. Univ. Alsk. Spec. Rep. 188–192.
