# Supplementary material for: Fecal glucocorticoid metabolites reflect hypothalamic–pituitary–adrenal axis activity in muskoxen (Ovibos moschatus)
Source: PLoS One. 2021 Apr 14;16(4):e0249281. doi: 10.1371/journal.pone.0249281 (PMC8046187; doi:10.1371/journal.pone.0249281)

S1 File: Results from the analytical validations of the cortisol and corticosterone enzyme immunoassays

Table 1: Polyclonal cortisol antibody R4866 cross-reactions (C. Munro, personal communication, 2010 (deceased in 2013))

**Steroid**	**% Cross Reaction**
Cortisol	100.0
Prednisolone	9.9
Prednisone	6.3
Compound S	6.2
Cortisone	5.0
Corticosterone	0.7
Desoxycorticosterone	0.3
21-desoxycortisone	0.5
11-desoxycortisol	0.2
Progesterone	0.2
17α-hydroxyprogesterone	0.2
Pregnenolone	0.1
17α-hydroxypregnenolone	0.1
Androstenedione	0.1
Testosterone	0.1
Androsterone	0.1
Dehyroepiandrosterone	0.1
Dehydroisoandrosterone-3-sulfate	0.1
Aldosterone	0.1
Estradiol-17β	0.1
Estrone	0.1
Estriol	0.1
Spironolactone	0.1
Cholesterol	0.1

Table 2: Polyclonal corticosterone antibody CJM006 cross-reactions (C. Munro, personal communication, 2010 (deceased in 2013))

**Steroid**	**% Cross Reaction**
Corticosterone	100.00
Desoxycorticosterone	14.25
Tetrahydrocorticosterone	0.90
11-Deoxycortisol	0.03
Prednisone	< 0.01
Prednisolone	0.07
Cortisol	0.23
Cortisone	< 0.01
Progesterone	2.65
Testosterone	0.64
Estradiol 17β	< 0.01

Figure 1: Serial dilutions showing parallel displacement with the standard curve for the cortisol antibody.

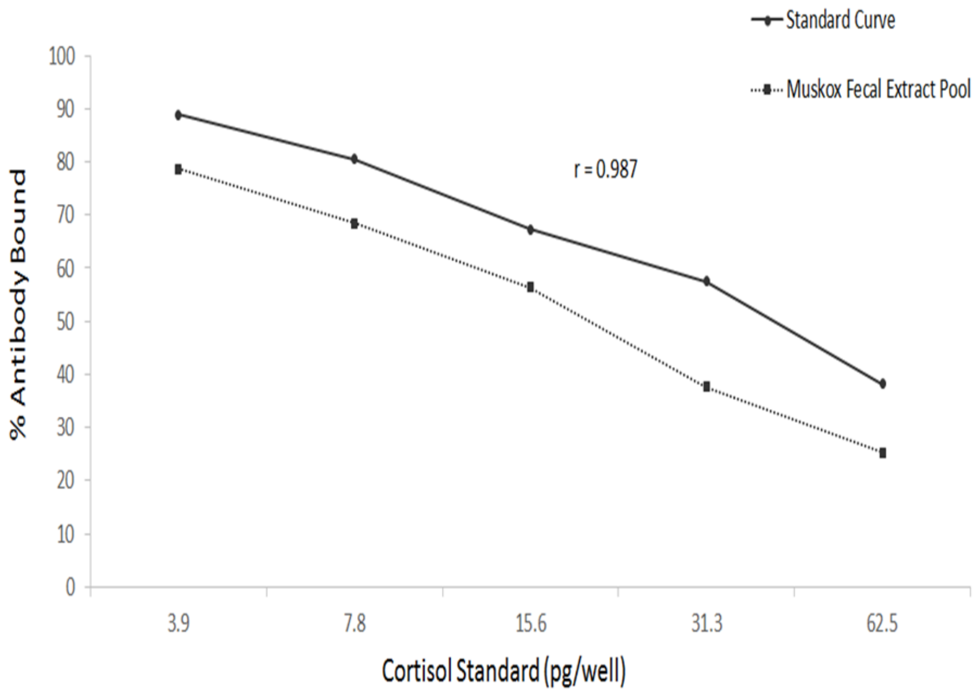

Figure 2: Serial dilutions showing parallel displacement with the standard curve for the corticosterone antibody.

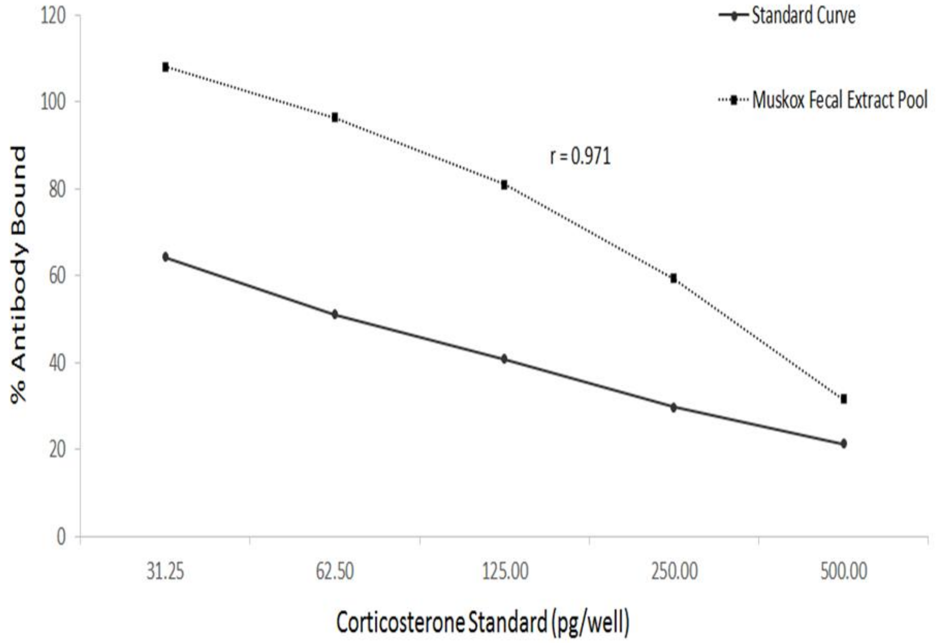

Figure 3: Recovery of exogenous cortisol added to a pooled muskox fecal extract. Samples were prepared as per protocol and spiked with cortisol standard at increasing concentrations.

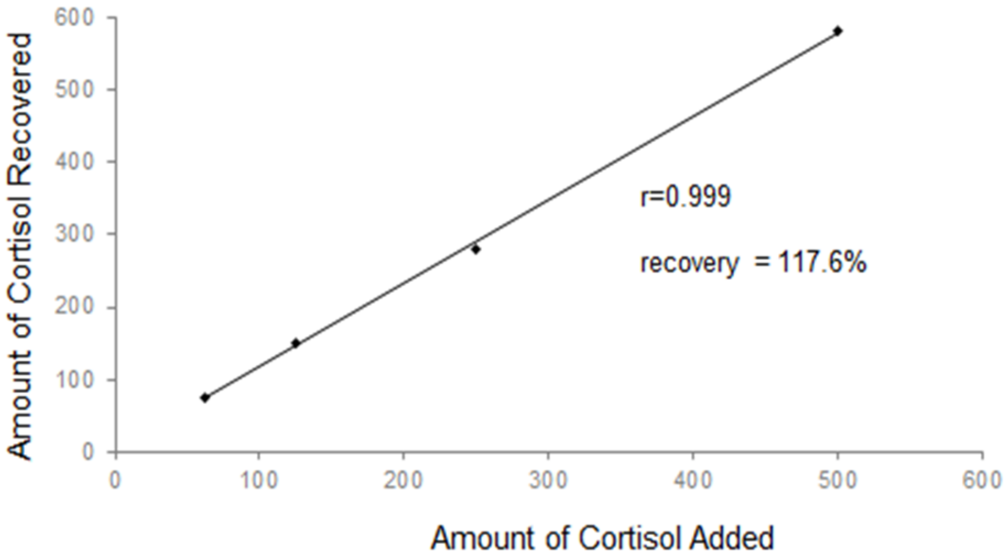

Figure 4: Recovery of exogenous corticosterone added to a pooled muskox fecal extract. Samples were prepared as per protocol and spiked with corticosterone standard at increasing concentrations.

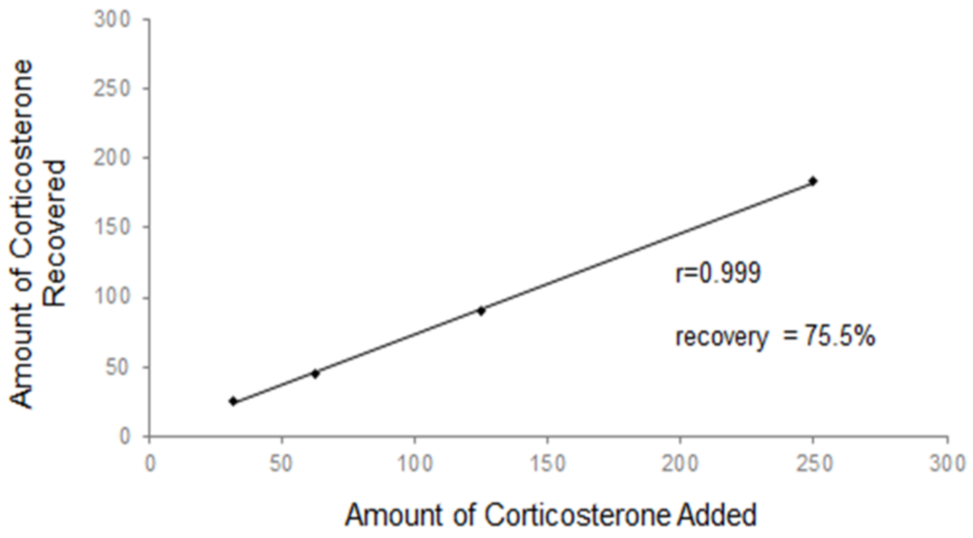

Supplement: S1 File — (PDF) [file pone.0249281.s003.pdf]
