# Supplementary material for: Fecal glucocorticoid metabolites reflect hypothalamic–pituitary–adrenal axis activity in muskoxen (Ovibos moschatus)
Source: PLoS One. 2021 Apr 14;16(4):e0249281. doi: 10.1371/journal.pone.0249281 (PMC8046187; doi:10.1371/journal.pone.0249281)

S2 File: **Fecal cortisol results**

Fecal cortisol levels were generally higher in the summer than in the winter, but exhibited important intra- and inter-individual variability and none of the animals showed a clear response following the ACTH injection during either of the challenges.

Table: Maximal percentage increase in fecal cortisol as compared to time 0 levels for the muskoxen given a single injection of ACTH (1 IU/kg) during the winter and/or a single injection of ACTH (2 IU/kg) or saline (control) during the summer, and the respective times post-injection at which it was observed.

	**Winter challenge**			**Summer challenge**		
Animal ID	Experimental group	Maximal percentage increase in fecal cortisol (%)	Time post-injection (h)	Experimental group	Maximal percentage increase in fecal cortisol	Time post-injection (h)
MX-738	/	/	/	ACTH	43	23
MX-740	ACTH	19	3	Control	118	8
MX-741	ACTH	28	25	ACTH	70	31
MX-620	ACTH	9	54	/	/	/
MX-621	ACTH	57	45	ACTH	0*	0*
MX-597	/	/	/	ACTH	41	25
MX-283	ACTH	30	95	Control	105	22
MX-1169	ACTH	69	5	ACTH	264	91

*The maximal fecal cortisol concentration was measured at time 0.

Figure: Individual fecal cortisol levels of the muskoxen as a function of the time following a single injection of ACTH during the winter (ACTH dose 1 IU/kg – n = 6) and/or of ACTH or saline (control) during the summer (ACTH dose 2 IU/kg – n = 5 ACTH-injected and n = 2 controls). Winter data are indicated as grey lines. Data for the ACTH-injected and control animals during the summer challenge correspond to the black and red lines, respectively.

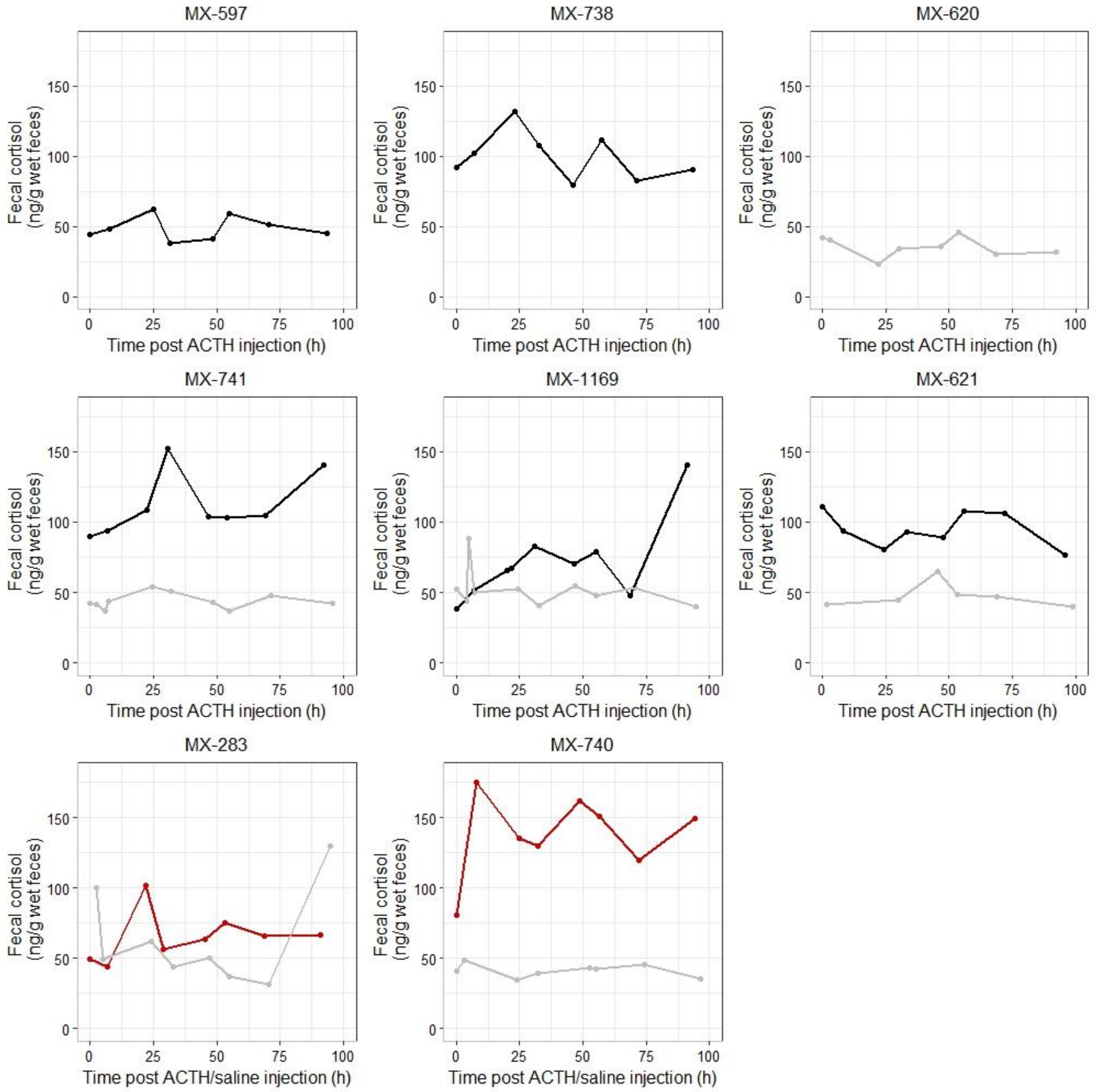

Supplement: S2 File — (PDF) [file pone.0249281.s004.pdf]
